# Supplementary material for: Human Metapneumovirus-Associated Hospitalization Burden in Children Aged Under 5 Years in Scotland Between 2017 and 2023: a Retrospective Analysis
Source: Open Forum Infect Dis. 2026 Aug 27;13(9):ofag532. doi: 10.1093/ofid/ofag532 (PMC13532078; doi:10.1093/ofid/ofag532)
Supplement: Supplementary file 1 [file ofag532_supplementary_data.docx]

Contents

[Supplementary Table S1: Underlying population characteristics in Lothian and other Scottish health boards in 2022 ^* †^	3](#_Toc233802457)

[Supplementary Table S2: ICD-10 respiratory tract infection diagnostic codes	4](#_Toc233802458)

[Supplementary Figure S1: Flow diagram describing the methods of estimating the laboratory-confirmed hospital incidence and extrapolated hospital incidence in Scotland	5](#_Toc233802459)

[Supplementary Figure S1a: Hospital incidence for Scotland based on laboratory-confirmed data from all Scottish health boards (without extrapolation)	5](#_Toc233802460)

[Supplementary Figure S1b: Hospital incidence for Scotland based on laboratory-confirmed data from Lothian and RTI hospital admissions in Scotland with extrapolation to health boards except Lothian	6](#_Toc233802461)

[Supplementary Text S1: Statistical analysis methods for extrapolation of hospital incidence	7](#_Toc233802462)

[Supplementary Table S3: Study population characteristics by health boards – Lothian and other Scottish health boards	9](#_Toc233802463)

[Supplementary Table S4: Laboratory-confirmed and extrapolated hospitalisation incidence of hMPV, RSV and Influenza A per 100,000 persons (with 95% CI) by age bands and season in <5y in Scotland	10](#_Toc233802464)

[Supplementary Table S5: Laboratory confirmed virus proportion positive in RTI admissions in Lothian by age bands and month	13](#_Toc233802465)

[Supplementary Table S6: Laboratory-confirmed and extrapolated hospital incidence per 100,000 persons (with 95% CI) of hMPV, RSV and Influenza A by age bands, season and health board (Lothian and other health boards) in older adults in Scotland	21](#_Toc233802466)

[Supplementary Table S7: Findings of sensitivity analysis conducted by limiting to episodes with specimens collected for viral testing between 3 days before and 3 days after the date of admission in older adults in Lothian by age bands and seasons	25](#_Toc233802467)

[Supplementary Table S8: Characteristics of all-cause RTI, hMPV, RSV, and Influenza A hospital episodes in older adults in Scotland (2017-2023)	27](#_Toc233802468)

[Supplementary Table S9: Number and proportion of laboratory-confirmed hMPV, RSV and Influenza A RTI episodes requiring long hospital length of stay (LOS) in children aged <5 years in Scotland by age bands during six annual seasons (2017-2023)	28](#_Toc233802469)

[Supplementary Table S10: ICU admission rate and in-hospital and post-discharge CFR associated with laboratory-confirmed hMPV, RSV, and Influenza A hospital admissions	29](#_Toc233802470)

# Supplementary Table S1: Underlying population characteristics in Lothian and other Scottish health boards in 2022 ^* †^

|  | **Lothian** | **Other Scottish health boards** | **Scotland** |
| --- | --- | --- | --- |
| **Total population (all ages)** | 904,628 | 4,535,214 | 5,439,842 |
| **0-5m** | 8,390 (0.93%) | 39,354 (0.87%) | 47,744 (0.88%) |
| **6m-1y** | 8,198 (0.91%) | 38,501 (0.85%) | 46,699 (0.86%) |
| **2-4y** | 25,626 (2.83%) | 128,336 (2.83%) | 153,962 (2.83%) |
| **Overall <5 years** | 42,214  (4.66%) | 206,191  (4.55%) | 248,405  (4.57%) |
| **White ethnicity** | 809,550  (89.49%) | 4,242,323  (93.5%) | 5,051,873  (92.87%) |
| **Percentage of health board population in the 10% most deprived areas (SIMD 2020)** | 4.2% | -- | 10% |

^*^National Records of Scotland. Ethnic group, national identity, language and religion 2025 [Available from: https://www.scotlandscensus.gov.uk/search-the-census#/topics/list?topic=Ethnic%20group,%20national%20identity,%20language%20and%20religion&categoryId=1

^†^ Public Health Scotland. Deprivation: data 2025 [Available from: <https://www.scotpho.org.uk/wider-determinants/deprivation/data/>.

Scottish Index of Multiple Deprivation (SIMD) ranks data zones in Scotland in order of deprivation. Lothian ranks 8th among 14 Scottish Health Boards for the proportion of its population in the 10% most deprived areas.

# Supplementary Table S2: ICD-10 respiratory tract infection diagnostic codes

| **Condition** | **ICD-10 codes** |
| --- | --- |
| Acute respiratory tract infections | J00\|J02\|J020\|J028\|J029\|J03\|J038\|J039\|J04\|J040\|J041\|J042\|J05\|J050\|J051\|J06\|J060\|J068\|J069\|J00\|J02\|J02.0\|J02.8\|J02.9\|J03\|J03.8\|J03.9\|J04\|J04.0\|J04.1\|J04.2\|J05\|J05.0\|J05.1\|J06\|J06.0\|J06.8\|J06.9 |
| Pneumonia and Influenza codes | J110\|J111\|J12\|J120\|J121\|J12\|J128\|J129\|J178\|J18\|J180\|J181\|J182\|J188\|J189\|J11.0\|J11.1\|J12\|J12.0\|J12.1\|J12\|J12.8\|J12.9\|J17.8\|J18\|J18.0\|J18.1\|J18.2\|J18.8\|J18.9 |
| Bronchiolitis and bronchitis | J20\|J200\|J201\|J202\|J206\|J207\|J208\|J209\|J21\|J218\|J219\|J40\|J20\|J20.0\|J20.1\|J20.2\|J20.6\|J20.7\|J20.8\|J20.9\|J21\|J21.8\|J21.9\|J40 |
| Unspecified LRTI | J22 |
| Human metapneumovirus codes | J123\|J211\|J12.3\|J21.1 |
| Respiratory syncytial virus codes | J121\|J205\|J210\|B974\|J12.1\|J20.5\|J21.0\|B97.4 |
| Flu codes | J09\|J10\|J100\|J101\|J108\|J11\|J11.8\|J09\|J10\|J10.0\|J10.1\|J10.8\|J11\|J11.8 |
| SARS-CoV-2 | U071\|U072\|U089\|U099\|U109\|U07.1\|U07.2\|U08.9\|U09.9\|U10.9 |

# Supplementary Figure S1: Flow diagram describing the methods of estimating the laboratory-confirmed hospital incidence and extrapolated hospital incidence in Scotland

## Supplementary Figure S1a: Hospital incidence for Scotland based on laboratory-confirmed data from all Scottish health boards (without extrapolation)

## Supplementary Figure S1b: Hospital incidence for Scotland based on laboratory-confirmed data from Lothian and RTI hospital admissions in Scotland with extrapolation to health boards except Lothian

|  | Data for Scotland (Lothian and other health boards) |
| --- | --- |
|  | Data for Lothian only |
|  | Data for health boards other than Lothian |

# Supplementary Text S1: Statistical analysis methods for extrapolation of hospital incidence

The detailed steps are described below:

1. Calculate the hMPV proportion positive among all RTI admissions in Lothian by age bands (0-5 months, 6m-1y and 2-4y), and month of admission during each annual season.

$$Lothian monthly hMPV in RTI proportion positive_{\left\{ ai, si, mi \right\}}=\frac{\{Lothian lab confirmed hMPV_{\left\{ ai,si, mi \right\}}\}}{\{Lothian all RTI hospital admissions_{\left\{ ai,si, mi \right\}}\}}$$

(where $a$ is the age band, $s$ is the annual season and $m$ is the month of admission)

2. Apply the Lothian hMPV proportion positive in RTI to the corresponding RTI admissions in other health boards to obtain the extrapolated hMPV-associated RTI admissions in other health boards by age band and month of admission during each annual season.

$${RoS extrapolated monthly hMPV RTI admissions}_{\left\{ ai, si,mi \right\}}=\left\{ Lothian monthly hMPV in RTI proportion positive_{\left\{ ai,si,mi \right\}} \right\} \left\{ RoS all RTI admissions_{\left\{ ai,si,mi \right\}} \right\}$$

(where $a$ is the age band, $s$ is the annual season and $m$ is the month of admission; RoS stands for rest of Scotland i.e., health boards except Lothian)

3. Sum up the monthly hMPV-associated RTI admissions in Lothian and other health boards to calculate the annual (July – June) hMPV-associated RTI admissions in Scotland according to age band.

$${Extrapolated annual hMPV RTI admissions in Scotland}_{\left\{ ai, si \right\}}=\sum_{m=July yi}^{June y(i+1)} \left( {Lothian lab confirmed hMPV}_{\left\{ ai, mi \right\}}+{RoS extrapolated monthly hMPV RTI admissions}_{\left\{ ai, mi \right\}} \right)$$

(where $a$ is the age band, $s$ is the annual season and $m$ is the month of admission; RoS stands for rest of Scotland i.e., health boards except Lothian)

4. Estimate the extrapolated annual hMPV-associated RTI hospital incidence in Scotland by age band.

$$Extrapolated annual hMPV RTI incidence_{\left\{ ai, si \right\}}= \frac{\left\{ Extrapolated annual hMP{V RTI admissions}_{\left\{ ai, si \right\}} \right\}\times100,000}{\{{Scottish population}_{\left\{ ai, si \right\}}\}}$$

(where $a$ is the age band and $s$ is the annual season)

5. Estimate the 95% confidence interval using non-parametric bootstrap resampling (1,000 replicates) based on grouped count (age band and season) and population data.

# Supplementary Table S3: Study population characteristics by health boards – Lothian and other Scottish health boards

|  |  | **All-cause RTI in Lothian**  **(n = 10,572)** | **All-cause RTI in other health boards**  **(n = 67,275)** | **Laboratory-confirmed hMPV in Lothian**  **(n = 505)** | **Laboratory-confirmed hMPV in other health boards**  **(n = 957)** | **Laboratory-confirmed RSV in Lothian**  **(n = 2,406)** | **Laboratory-confirmed RSV in other health boards**  **(n = 8,579)** | **Laboratory-confirmed Influenza A in Lothian**  **(n = 377)** | **Laboratory-confirmed Influenza A in other health boards**  **(n = 1,747)** |
| --- | --- | --- | --- | --- | --- | --- | --- | --- | --- |
| **Age ^[1]^** | 0-5 months | 3,242 (30.7%) | 16,515  (24.5%) | 122  (24.2%) | 196 (20.5%) | 1,101 (45.8%) | 3,924 (45.7%) | 68  (18%) | 253 (14.5%) |
|  | 6 months – 1 year | 4,723  (44.7%) | 31,395  (46.7%) | 230 (45.5%) | 464 (48.5%) | 971 (40.4%) | 3,324 (38.7%) | 156 (41.4%) | 735 (42.1%) |
|  | 2-4 years | 2607  (24.7%) | 193,65  (28.8%) | 153 (30.3%) | 297  (31%) | 334 (13.9%) | 1,331 (15.5%) | 153 (40.6%) | 759 (43.4%) |
| **Sex** | Female | 4,442  (42%) | 28,228  (42%) | 209 (41.4%) | 406 (42.4%) | 1,072 (44.6%) | 3,795 (44.2%) | 157 (41.6%) | 792 (45.3%) |
|  | Male | 6,130  (58%) | 39,047  (58%) | 296  (58.6%) | 551 (57.6%) | 1,334 (55.4%) | 4,784 (55.8%) | 220 (58.4%) | 955 (54.7%) |
| **Ethnicity ^[2]^** | White | 8,388  (79.3%) | 47,124  (70%) | 408 (80.8%) | 677 (70.7%) | 1,892 (78.6%) | 5,649  (65.8%) | 287  (76.1%) | 1237 (70.8%) |
|  | Non-White | 2,184  (21.7%) | 16,286  (24.2%) | 97  (19.2%) | 253 (26.4%) | 514  (21.4%) | 2,106  (24.5%) | 90 (23.9%) | 381 (21.8%) |
|  | Data unavailable | 0 | 3,865  (5.7%) | 0 | 27  (2.8%) | 0 | 824  (9.6%) | 0 | 129 (7.4%) |
| **SIMD ^[3]^** | 1 | 1,937 (18.3%) | 19,916 (29.6%) | 100 (19.8%) | 219  (22.9%) | 438  (18.2%) | 2,498 (29.1%) | 85 (22.5%) | 536  (30.7%) |
|  | 2 | 2,585 (24.5%) | 14,234  (21.2%) | 113 (22.4%) | 208  (21.7%) | 614 (25.5%) | 1,721  (20.1%) | 86 (22.8%) | 392  (22.4%) |
|  | 3 | 1,669 (15.8%) | 12,016 (17.9%) | 86 (17.0%) | 202  (21.1%) | 372 (15.5%) | 1,614  (18.8%) | 53 (14.1%) | 311 (17.8%) |
|  | 4 | 2,291 (21.7%) | 11,632 (17.3%) | 102 (20.2%) | 173  (18.1%) | 520 (21.6%) | 1,516  (17.7%) | 77 (20.4%) | 277 (15.9%) |
|  | 5 | 2,065 (19.5%) | 9,076 (13.5%) | 104  (20.6%) | 151  (15.8%) | 457  (19%) | 1,184  (13.8%) | 75 (19.9%) | 221 (12.7%) |
|  | Data unavailable | 25 (0.2%) | 401 (0.6%) | 0 | 4  (0.4%) | 5  (0.2%) | 46  (0.5%) | 1 (0.3%) | 10 (0.6%) |

# Supplementary Table S4: Laboratory-confirmed and extrapolated hospitalisation incidence of hMPV, RSV and Influenza A per 100,000 persons (with 95% CI) by age bands and season in <5y in Scotland

| **Season** | **Age band** | **All-cause RTI hospital incidence** | **Lab-confirmed hMPV hospital incidence** | **Extrapolated hMPV hospital incidence** | **Lab-confirmed RSV hospital incidence** | **Extrapolated RSV hospital incidence** | **Lab-confirmed Influenza A hospital incidence** | **Extrapolated Influenza A hospital incidence** |
| --- | --- | --- | --- | --- | --- | --- | --- | --- |
| 2017/18 | 0-5m | 7228.3 (7017.0 - 7461.0) | 108.6 (81.4 - 135.7) | 180.4 (143.5 - 217.2) | 1696.6 (1584.1 - 1809.1) | 2238.8 (2115.3 - 2367.5) | 164.8 (129.9 - 197.8) | 313.1 (265.6 - 364.5) |
| 2018/19 | 0-5m | 7762.6 (7537.5 - 7987.7) | 150.0 (116.4 - 183.6) | 396.5 (341.4 - 454.0) | 1734.9 (1626.3 - 1855.3) | 2802.8 (2664.5 - 2948.8) | 171.7 (134.2 - 211.2) | 211.1 (169.7 - 250.7) |
| 2019/20 | 0-5m | 7641.7 (7412.5 - 7883.2) | 99.1 (68.1 - 128) | 264.6 (218.8 - 313.8) | 2470.8 (2336.7 - 2613.3) | 3243.5 (3085.9 - 3397.7) | 177.5 (138.3 - 218.8) | 270.6 (227.1 - 320.0) |
| 2020/21 | 0-5m | 2122.1 (1993.6 - 2248.4) | 8.6 (2.1 - 19.3) | ^[4]^ | 2.1 (0 - 6.4) | * | 2.1 (0 - 6.4) | * |
| 2021/22 | 0-5m | 10247.7 (9997.6 - 10525.3) | 254.3 (209.8 - 298.8) | 666.9 (595.5 - 741.7) | 2316.1 (2180.5 - 2434.8) | 3425.9 (3261.2 - 3598.1) | 38.1 (21.2 - 55.1) | 47.5 (29.6 - 67.9) |
| 2022/23 | 0-5m | 5556.6 (5363.8 - 5764.6) | 30.3 (17.3 - 47.6) | 31.4 (17.3 - 47.6) | 2121.3 (1991.4 - 2262.1) | 1878.6 (1751.2 - 2011.0) | 95.2 (69.3 - 123.4) | 92.2 (64.9 - 121.2) |
| 2017/18 | 6m-1y | 13855.9 (13573.6 - 14157.0) | 231.0 (189.4 - 272.7) | 646.6 (579.5 - 719.6) | 1374.8 (1270.6 - 1473.3) | 2233.8 (2105.7 - 2367.1) | 321.9 (274.5 - 369.3) | 457.4 (397.7 - 515.1) |
| 2018/19 | 6m-1y | 13771.9 (13484.3 - 14078.7) | 256.6 (214.2 - 303.0) | 790.2 (717.8 - 874.1) | 1574.6 (1466.5 - 1678.9) | 3284.9 (3131.8 - 3450.3) | 553.8 (490.1 - 617.5) | 944.8 (866.4 - 1030.4) |
| 2019/20 | 6m-1y | 13102.6 (12800.5 - 13408.6) | 192.2 (154.9 - 231.4) | 432.3 (378.5 - 488.3) | 2153.4 (2033.7 - 2282.9) | 3791.0 (3622.3 - 3971.4) | 423.6 (368.7 - 482.4) | 558.9 (494.2 - 625.7) |
| 2020/21 | 6m-1y | 6460.7 (6240.6 - 6678.7) | 22.6 (10.3 - 37.0) | 56.5 (37.0 - 76.2) | 8.2 (2.1 - 18.5) | * | 2.1 (0 - 6.2) | * |
| 2021/22 | 6m-1y | 15181.9 (14879.5 - 15511.8) | 539.6 (474.5 - 606.8) | 1201.4 (1102.3 - 1299.6) | 1725.8 (1618.7 - 1837.2) | 3289.3 (3136.7 - 3445.5) | 79.8 (54.6 - 105.0) | 104.6 (75.6 - 136.5) |
| 2022/23 | 6m-1y | 9512.1 (9256.3 - 9774.2) | 150.6 (115.5 - 183.6) | 374.1 (321.8 - 431.2) | 1710.2 (1586.3 - 1821.6) | 1874.7 (1763.8 - 2001.1) | 369.3 (315.6 - 422.9) | 394.5 (342.4 - 453.8) |
| 2017/18 | 2-4y | 2331.6 (2259.3 - 2404.0) | 41.3 (31.7 - 52.0) | 129.9 (114.2 - 147.7) | 135.1 (117.8 - 153.1) | 199.2 (177.0 - 223.6) | 76.5 (64.0 - 90.3) | 106.7 (90.9 - 123.2) |
| 2018/19 | 2-4y | 2519.4 (2442.8 - 2593.0) | 48.0 (37.7 - 59.0) | 168.0 (148.3 - 188.5) | 150.8 (132.5 - 170.8) | 338.7 (313.1 - 366.6) | 187.9 (166.0 - 210.4) | 354.1 (325.3 - 382.4) |
| 2019/20 | 2-4y | 2353.2 (2278.2 - 2425.8) | 36.9 (28.1 - 45.7) | 97.1 (81.3 - 112.6) | 193.9 (173.9 - 215.8) | 270.1 (244.5 - 297.7) | 115.7 (98.2 - 132.0) | 176.7 (155.7 - 198.3) |
| 2020/21 | 2-4y | 1120.5 (1072.3 - 1175.1) | 5.1 (1.9 - 9.0) | 10.7 (5.8 - 16.1) | 2.6 (0.6 - 5.1) | 7.5 (3.2 - 12.2) | 2.6 (0.6 - 5.1) | * |
| 2021/22 | 2-4y | 3256.2 (3165.3 - 3343.3) | 119.0 (101.4 - 136.7) | 290.0 (262.2 - 315.9) | 329.6 (302.1 - 358.4) | 552.8 (514.0 - 594.5) | 40.5 (31.4 - 50.4) | 60.5 (48.4 - 73.9) |
| 2022/23 | 2-4y | 2255.6 (2179.6 - 2327.7) | 34.7 (25.6 - 44.6) | 100.5 (85.2 - 116.6) | 244.4 (220.8 - 268.7) | 319.1 (290.3 - 346.7) | 146.8 (127.1 - 166.5) | 170.4 (150.1 - 190.7) |
| 2017/18 | <5y | 5501.6 (5417.7 - 5588.2) | 90.9 (79.9 - 102.3) | 240.0 (221.6 - 258.8) | 672.6 (642.0 - 702.0) | 982.0 (943.9 - 1020.1) | 141.0 (127.0 - 155.7) | 214 (196.6 - 231.9) |
| 2018/19 | <5y | 5698.7 (5614.4 - 5786.4) | 107.9 (95.1 - 120.2) | 332.2 (312.0 - 354.7) | 727.8 (694.1 - 762.2) | 1378.2 (1336.1 - 1420.4) | 255.8 (237.1 - 275.7) | 441.6 (418.8 - 467.8) |
| 2019/20 | <5y | 5454.6 (5363.5 - 5539.0) | 79.0 (68.2 - 89.1) | 194.3 (178.5 - 211.3) | 1004.5 (965.9 - 1044.2) | 1517.8 (1467.9 - 1564.0) | 187.8 (170.0 - 204.7) | 269.4 (248.3 - 288.8) |
| 2020/21 | <5y | 2341.4 (2284.0 - 2403.2) | 9.2 (6.0 - 13.1) | 19.2 (13.9 - 24.7) | 3.6 (1.6 - 6.0) | 6.6 (3.6 - 10.0) | 2.4 (0.8 - 4.4) | * |
| 2021/22 | <5y | 6881.0 (6784.1 - 6980.7) | 225.7 (205.9 - 244.2) | 537.0 (507.0 - 566.0) | 976.5 (936.1 - 1012.8) | 1626.3 (1576.3 - 1672.0) | 47.6 (40.0 - 56.9) | 66.5 (56.5 - 76.7) |
| 2022/23 | <5y | 4294.9 (4216.5 - 4371.0) | 56.6 (47.7 - 66.3) | 141.2 (127.8 - 155.7) | 882.4 (844.8 - 918.4) | 915.4 (880.4 - 953.6) | 180.8 (163.0 - 197.4) | 199.7 (182.8 - 217.2) |

# Supplementary Table S5: Laboratory confirmed virus proportion positive in RTI admissions in Lothian by age bands and month

| **Virus** | **Age band** | **Virus proportion positive in RTI episodes** | **Month and year of hospital admission** |
| --- | --- | --- | --- |
| hMPV | 0-5m | 0.011 | November 2017 |
| hMPV | 0-5m | 0.030 | January 2018 |
| hMPV | 0-5m | 0.054 | February 2018 |
| hMPV | 0-5m | 0.027 | March 2018 |
| hMPV | 0-5m | 0.148 | April 2018 |
| hMPV | 0-5m | 0.043 | May 2018 |
| hMPV | 0-5m | 0.059 | August 2018 |
| hMPV | 0-5m | 0.032 | October 2018 |
| hMPV | 0-5m | 0.037 | November 2018 |
| hMPV | 0-5m | 0.052 | December 2018 |
| hMPV | 0-5m | 0.112 | January 2019 |
| hMPV | 0-5m | 0.057 | February 2019 |
| hMPV | 0-5m | 0.061 | March 2019 |
| hMPV | 0-5m | 0.059 | April 2019 |
| hMPV | 0-5m | 0.030 | May 2019 |
| hMPV | 0-5m | 0.071 | June 2019 |
| hMPV | 0-5m | 0.143 | August 2019 |
| hMPV | 0-5m | 0.028 | September 2019 |
| hMPV | 0-5m | 0.021 | October 2019 |
| hMPV | 0-5m | 0.006 | November 2019 |
| hMPV | 0-5m | 0.049 | December 2019 |
| hMPV | 0-5m | 0.100 | January 2020 |
| hMPV | 0-5m | 0.083 | February 2020 |
| hMPV | 0-5m | 0.032 | March 2020 |
| hMPV | 0-5m | 0.048 | July 2021 |
| hMPV | 0-5m | 0.020 | September 2021 |
| hMPV | 0-5m | 0.030 | October 2021 |
| hMPV | 0-5m | 0.210 | November 2021 |
| hMPV | 0-5m | 0.317 | December 2021 |
| hMPV | 0-5m | 0.073 | January 2022 |
| hMPV | 0-5m | 0.019 | April 2022 |
| hMPV | 0-5m | 0.009 | October 2022 |
| hMPV | 0-5m | 0.050 | December 2022 |
| hMPV | 6m-1y | 0.053 | July 2017 |
| hMPV | 6m-1y | 0.013 | September 2017 |
| hMPV | 6m-1y | 0.065 | November 2017 |
| hMPV | 6m-1y | 0.054 | December 2017 |
| hMPV | 6m-1y | 0.026 | January 2018 |
| hMPV | 6m-1y | 0.089 | February 2018 |
| hMPV | 6m-1y | 0.044 | March 2018 |
| hMPV | 6m-1y | 0.056 | April 2018 |
| hMPV | 6m-1y | 0.109 | May 2018 |
| hMPV | 6m-1y | 0.037 | June 2018 |
| hMPV | 6m-1y | 0.095 | July 2018 |
| hMPV | 6m-1y | 0.032 | November 2018 |
| hMPV | 6m-1y | 0.068 | December 2018 |
| hMPV | 6m-1y | 0.127 | January 2019 |
| hMPV | 6m-1y | 0.081 | February 2019 |
| hMPV | 6m-1y | 0.115 | March 2019 |
| hMPV | 6m-1y | 0.029 | April 2019 |
| hMPV | 6m-1y | 0.049 | May 2019 |
| hMPV | 6m-1y | 0.036 | June 2019 |
| hMPV | 6m-1y | 0.014 | September 2019 |
| hMPV | 6m-1y | 0.033 | October 2019 |
| hMPV | 6m-1y | 0.031 | November 2019 |
| hMPV | 6m-1y | 0.030 | December 2019 |
| hMPV | 6m-1y | 0.118 | January 2020 |
| hMPV | 6m-1y | 0.048 | February 2020 |
| hMPV | 6m-1y | 0.032 | March 2020 |
| hMPV | 6m-1y | 0.091 | August 2020 |
| hMPV | 6m-1y | 0.011 | August 2021 |
| hMPV | 6m-1y | 0.019 | October 2021 |
| hMPV | 6m-1y | 0.266 | November 2021 |
| hMPV | 6m-1y | 0.342 | December 2021 |
| hMPV | 6m-1y | 0.097 | January 2022 |
| hMPV | 6m-1y | 0.034 | February 2022 |
| hMPV | 6m-1y | 0.008 | October 2022 |
| hMPV | 6m-1y | 0.026 | November 2022 |
| hMPV | 6m-1y | 0.197 | December 2022 |
| hMPV | 6m-1y | 0.062 | January 2023 |
| hMPV | 6m-1y | 0.250 | February 2023 |
| hMPV | 6m-1y | 0.022 | March 2023 |
| hMPV | 6m-1y | 0.056 | April 2023 |
| hMPV | 6m-1y | 0.059 | May 2023 |
| hMPV | 2-4y | 0.133 | July 2017 |
| hMPV | 2-4y | 0.020 | November 2017 |
| hMPV | 2-4y | 0.081 | December 2017 |
| hMPV | 2-4y | 0.118 | January 2018 |
| hMPV | 2-4y | 0.077 | February 2018 |
| hMPV | 2-4y | 0.030 | March 2018 |
| hMPV | 2-4y | 0.056 | April 2018 |
| hMPV | 2-4y | 0.091 | May 2018 |
| hMPV | 2-4y | 0.053 | June 2018 |
| hMPV | 2-4y | 0.133 | October 2018 |
| hMPV | 2-4y | 0.038 | November 2018 |
| hMPV | 2-4y | 0.066 | December 2018 |
| hMPV | 2-4y | 0.082 | January 2019 |
| hMPV | 2-4y | 0.114 | March 2019 |
| hMPV | 2-4y | 0.182 | April 2019 |
| hMPV | 2-4y | 0.133 | May 2019 |
| hMPV | 2-4y | 0.143 | June 2019 |
| hMPV | 2-4y | 0.026 | July 2019 |
| hMPV | 2-4y | 0.031 | August 2019 |
| hMPV | 2-4y | 0.057 | September 2019 |
| hMPV | 2-4y | 0.026 | October 2019 |
| hMPV | 2-4y | 0.016 | November 2019 |
| hMPV | 2-4y | 0.011 | December 2019 |
| hMPV | 2-4y | 0.056 | January 2020 |
| hMPV | 2-4y | 0.133 | February 2020 |
| hMPV | 2-4y | 0.088 | March 2020 |
| hMPV | 2-4y | 0.111 | April 2020 |
| hMPV | 2-4y | 0.023 | June 2021 |
| hMPV | 2-4y | 0.036 | July 2021 |
| hMPV | 2-4y | 0.022 | September 2021 |
| hMPV | 2-4y | 0.089 | October 2021 |
| hMPV | 2-4y | 0.200 | November 2021 |
| hMPV | 2-4y | 0.325 | December 2021 |
| hMPV | 2-4y | 0.156 | January 2022 |
| hMPV | 2-4y | 0.040 | February 2022 |
| hMPV | 2-4y | 0.043 | March 2022 |
| hMPV | 2-4y | 0.041 | September 2022 |
| hMPV | 2-4y | 0.016 | October 2022 |
| hMPV | 2-4y | 0.108 | November 2022 |
| hMPV | 2-4y | 0.044 | December 2022 |
| hMPV | 2-4y | 0.136 | January 2023 |
| hMPV | 2-4y | 0.130 | February 2023 |
| hMPV | 2-4y | 0.050 | March 2023 |
| RSV | 0-5m | 0.053 | July 2017 |
| RSV | 0-5m | 0.133 | August 2017 |
| RSV | 0-5m | 0.115 | September 2017 |
| RSV | 0-5m | 0.314 | October 2017 |
| RSV | 0-5m | 0.534 | November 2017 |
| RSV | 0-5m | 0.542 | December 2017 |
| RSV | 0-5m | 0.197 | January 2018 |
| RSV | 0-5m | 0.081 | February 2018 |
| RSV | 0-5m | 0.081 | March 2018 |
| RSV | 0-5m | 0.111 | April 2018 |
| RSV | 0-5m | 0.087 | May 2018 |
| RSV | 0-5m | 0.300 | June 2018 |
| RSV | 0-5m | 0.059 | August 2018 |
| RSV | 0-5m | 0.219 | September 2018 |
| RSV | 0-5m | 0.419 | October 2018 |
| RSV | 0-5m | 0.670 | November 2018 |
| RSV | 0-5m | 0.607 | December 2018 |
| RSV | 0-5m | 0.325 | January 2019 |
| RSV | 0-5m | 0.132 | February 2019 |
| RSV | 0-5m | 0.041 | March 2019 |
| RSV | 0-5m | 0.029 | April 2019 |
| RSV | 0-5m | 0.059 | July 2019 |
| RSV | 0-5m | 0.190 | August 2019 |
| RSV | 0-5m | 0.250 | September 2019 |
| RSV | 0-5m | 0.562 | October 2019 |
| RSV | 0-5m | 0.632 | November 2019 |
| RSV | 0-5m | 0.504 | December 2019 |
| RSV | 0-5m | 0.400 | January 2020 |
| RSV | 0-5m | 0.125 | February 2020 |
| RSV | 0-5m | 0.143 | July 2021 |
| RSV | 0-5m | 0.641 | August 2021 |
| RSV | 0-5m | 0.741 | September 2021 |
| RSV | 0-5m | 0.570 | October 2021 |
| RSV | 0-5m | 0.129 | November 2021 |
| RSV | 0-5m | 0.098 | December 2021 |
| RSV | 0-5m | 0.055 | January 2022 |
| RSV | 0-5m | 0.068 | March 2022 |
| RSV | 0-5m | 0.074 | April 2022 |
| RSV | 0-5m | 0.133 | May 2022 |
| RSV | 0-5m | 0.329 | June 2022 |
| RSV | 0-5m | 0.115 | July 2022 |
| RSV | 0-5m | 0.169 | August 2022 |
| RSV | 0-5m | 0.444 | September 2022 |
| RSV | 0-5m | 0.440 | October 2022 |
| RSV | 0-5m | 0.477 | November 2022 |
| RSV | 0-5m | 0.250 | December 2022 |
| RSV | 0-5m | 0.375 | January 2023 |
| RSV | 6m-1y | 0.026 | July 2017 |
| RSV | 6m-1y | 0.065 | August 2017 |
| RSV | 6m-1y | 0.080 | September 2017 |
| RSV | 6m-1y | 0.228 | October 2017 |
| RSV | 6m-1y | 0.336 | November 2017 |
| RSV | 6m-1y | 0.369 | December 2017 |
| RSV | 6m-1y | 0.077 | January 2018 |
| RSV | 6m-1y | 0.044 | February 2018 |
| RSV | 6m-1y | 0.015 | March 2018 |
| RSV | 6m-1y | 0.056 | April 2018 |
| RSV | 6m-1y | 0.036 | May 2018 |
| RSV | 6m-1y | 0.037 | June 2018 |
| RSV | 6m-1y | 0.143 | July 2018 |
| RSV | 6m-1y | 0.075 | August 2018 |
| RSV | 6m-1y | 0.089 | September 2018 |
| RSV | 6m-1y | 0.459 | October 2018 |
| RSV | 6m-1y | 0.529 | November 2018 |
| RSV | 6m-1y | 0.444 | December 2018 |
| RSV | 6m-1y | 0.203 | January 2019 |
| RSV | 6m-1y | 0.058 | February 2019 |
| RSV | 6m-1y | 0.090 | March 2019 |
| RSV | 6m-1y | 0.055 | June 2019 |
| RSV | 6m-1y | 0.065 | July 2019 |
| RSV | 6m-1y | 0.122 | August 2019 |
| RSV | 6m-1y | 0.229 | September 2019 |
| RSV | 6m-1y | 0.496 | October 2019 |
| RSV | 6m-1y | 0.556 | November 2019 |
| RSV | 6m-1y | 0.351 | December 2019 |
| RSV | 6m-1y | 0.197 | January 2020 |
| RSV | 6m-1y | 0.048 | February 2020 |
| RSV | 6m-1y | 0.016 | March 2020 |
| RSV | 6m-1y | 0.096 | July 2021 |
| RSV | 6m-1y | 0.521 | August 2021 |
| RSV | 6m-1y | 0.559 | September 2021 |
| RSV | 6m-1y | 0.330 | October 2021 |
| RSV | 6m-1y | 0.032 | November 2021 |
| RSV | 6m-1y | 0.017 | December 2021 |
| RSV | 6m-1y | 0.014 | January 2022 |
| RSV | 6m-1y | 0.069 | February 2022 |
| RSV | 6m-1y | 0.045 | March 2022 |
| RSV | 6m-1y | 0.180 | April 2022 |
| RSV | 6m-1y | 0.203 | May 2022 |
| RSV | 6m-1y | 0.198 | June 2022 |
| RSV | 6m-1y | 0.158 | July 2022 |
| RSV | 6m-1y | 0.189 | August 2022 |
| RSV | 6m-1y | 0.202 | September 2022 |
| RSV | 6m-1y | 0.297 | October 2022 |
| RSV | 6m-1y | 0.368 | November 2022 |
| RSV | 6m-1y | 0.098 | December 2022 |
| RSV | 6m-1y | 0.094 | January 2023 |
| RSV | 6m-1y | 0.022 | March 2023 |
| RSV | 6m-1y | 0.111 | April 2023 |
| RSV | 6m-1y | 0.118 | May 2023 |
| RSV | 2-4y | 0.100 | October 2017 |
| RSV | 2-4y | 0.327 | November 2017 |
| RSV | 2-4y | 0.145 | December 2017 |
| RSV | 2-4y | 0.059 | January 2018 |
| RSV | 2-4y | 0.030 | March 2018 |
| RSV | 2-4y | 0.036 | September 2018 |
| RSV | 2-4y | 0.300 | October 2018 |
| RSV | 2-4y | 0.410 | November 2018 |
| RSV | 2-4y | 0.355 | December 2018 |
| RSV | 2-4y | 0.020 | January 2019 |
| RSV | 2-4y | 0.075 | September 2019 |
| RSV | 2-4y | 0.224 | October 2019 |
| RSV | 2-4y | 0.317 | November 2019 |
| RSV | 2-4y | 0.126 | December 2019 |
| RSV | 2-4y | 0.023 | June 2021 |
| RSV | 2-4y | 0.036 | July 2021 |
| RSV | 2-4y | 0.611 | August 2021 |
| RSV | 2-4y | 0.516 | September 2021 |
| RSV | 2-4y | 0.107 | October 2021 |
| RSV | 2-4y | 0.012 | November 2021 |
| RSV | 2-4y | 0.013 | December 2021 |
| RSV | 2-4y | 0.020 | February 2022 |
| RSV | 2-4y | 0.022 | March 2022 |
| RSV | 2-4y | 0.139 | April 2022 |
| RSV | 2-4y | 0.157 | May 2022 |
| RSV | 2-4y | 0.179 | June 2022 |
| RSV | 2-4y | 0.205 | July 2022 |
| RSV | 2-4y | 0.130 | August 2022 |
| RSV | 2-4y | 0.178 | September 2022 |
| RSV | 2-4y | 0.219 | October 2022 |
| RSV | 2-4y | 0.169 | November 2022 |
| RSV | 2-4y | 0.067 | December 2022 |
| RSV | 2-4y | 0.045 | January 2023 |
| RSV | 2-4y | 0.043 | February 2023 |
| RSV | 2-4y | 0.150 | March 2023 |
| RSV | 2-4y | 0.125 | April 2023 |
| RSV | 2-4y | 0.111 | June 2023 |
| Influenza A | 0-5m | 0.038 | September 2017 |
| Influenza A | 0-5m | 0.029 | October 2017 |
| Influenza A | 0-5m | 0.011 | November 2017 |
| Influenza A | 0-5m | 0.084 | December 2017 |
| Influenza A | 0-5m | 0.106 | January 2018 |
| Influenza A | 0-5m | 0.027 | March 2018 |
| Influenza A | 0-5m | 0.074 | April 2018 |
| Influenza A | 0-5m | 0.007 | December 2018 |
| Influenza A | 0-5m | 0.100 | January 2019 |
| Influenza A | 0-5m | 0.094 | February 2019 |
| Influenza A | 0-5m | 0.061 | March 2019 |
| Influenza A | 0-5m | 0.048 | August 2019 |
| Influenza A | 0-5m | 0.028 | September 2019 |
| Influenza A | 0-5m | 0.010 | October 2019 |
| Influenza A | 0-5m | 0.011 | November 2019 |
| Influenza A | 0-5m | 0.081 | December 2019 |
| Influenza A | 0-5m | 0.083 | January 2020 |
| Influenza A | 0-5m | 0.018 | January 2022 |
| Influenza A | 0-5m | 0.018 | October 2022 |
| Influenza A | 0-5m | 0.023 | November 2022 |
| Influenza A | 0-5m | 0.050 | December 2022 |
| Influenza A | 0-5m | 0.125 | January 2023 |
| Influenza A | 6m-1y | 0.032 | August 2017 |
| Influenza A | 6m-1y | 0.009 | November 2017 |
| Influenza A | 6m-1y | 0.077 | December 2017 |
| Influenza A | 6m-1y | 0.141 | January 2018 |
| Influenza A | 6m-1y | 0.044 | March 2018 |
| Influenza A | 6m-1y | 0.013 | November 2018 |
| Influenza A | 6m-1y | 0.060 | December 2018 |
| Influenza A | 6m-1y | 0.271 | January 2019 |
| Influenza A | 6m-1y | 0.221 | February 2019 |
| Influenza A | 6m-1y | 0.077 | March 2019 |
| Influenza A | 6m-1y | 0.031 | November 2019 |
| Influenza A | 6m-1y | 0.134 | December 2019 |
| Influenza A | 6m-1y | 0.092 | January 2020 |
| Influenza A | 6m-1y | 0.012 | February 2020 |
| Influenza A | 6m-1y | 0.016 | March 2020 |
| Influenza A | 6m-1y | 0.060 | April 2022 |
| Influenza A | 6m-1y | 0.012 | June 2022 |
| Influenza A | 6m-1y | 0.011 | August 2022 |
| Influenza A | 6m-1y | 0.008 | October 2022 |
| Influenza A | 6m-1y | 0.079 | November 2022 |
| Influenza A | 6m-1y | 0.213 | December 2022 |
| Influenza A | 6m-1y | 0.062 | January 2023 |
| Influenza A | 6m-1y | 0.062 | February 2023 |
| Influenza A | 2-4y | 0.067 | July 2017 |
| Influenza A | 2-4y | 0.020 | November 2017 |
| Influenza A | 2-4y | 0.129 | December 2017 |
| Influenza A | 2-4y | 0.147 | January 2018 |
| Influenza A | 2-4y | 0.026 | February 2018 |
| Influenza A | 2-4y | 0.030 | March 2018 |
| Influenza A | 2-4y | 0.028 | April 2018 |
| Influenza A | 2-4y | 0.045 | May 2018 |
| Influenza A | 2-4y | 0.033 | October 2018 |
| Influenza A | 2-4y | 0.051 | November 2018 |
| Influenza A | 2-4y | 0.118 | December 2018 |
| Influenza A | 2-4y | 0.367 | January 2019 |
| Influenza A | 2-4y | 0.442 | February 2019 |
| Influenza A | 2-4y | 0.114 | March 2019 |
| Influenza A | 2-4y | 0.030 | April 2019 |
| Influenza A | 2-4y | 0.036 | June 2019 |
| Influenza A | 2-4y | 0.032 | November 2019 |
| Influenza A | 2-4y | 0.253 | December 2019 |
| Influenza A | 2-4y | 0.111 | January 2020 |
| Influenza A | 2-4y | 0.033 | February 2020 |
| Influenza A | 2-4y | 0.088 | March 2020 |
| Influenza A | 2-4y | 0.018 | October 2021 |
| Influenza A | 2-4y | 0.013 | December 2021 |
| Influenza A | 2-4y | 0.020 | February 2022 |
| Influenza A | 2-4y | 0.043 | March 2022 |
| Influenza A | 2-4y | 0.111 | April 2022 |
| Influenza A | 2-4y | 0.020 | May 2022 |
| Influenza A | 2-4y | 0.043 | August 2022 |
| Influenza A | 2-4y | 0.014 | September 2022 |
| Influenza A | 2-4y | 0.047 | October 2022 |
| Influenza A | 2-4y | 0.046 | November 2022 |
| Influenza A | 2-4y | 0.356 | December 2022 |
| Influenza A | 2-4y | 0.182 | January 2023 |

# Supplementary Table S6: Laboratory-confirmed and extrapolated hospital incidence per 100,000 persons (with 95% CI) of hMPV, RSV and Influenza A by age bands, season and health board (Lothian and other health boards) in older adults in Scotland

| **Season** | **Age band** | **Region** | **All-cause RTI incidence** | **Lab confirmed hMPV incidence** | **Extrapolated hMPV incidence** | **Lab confirmed RSV incidence** | **Extrapolated RSV incidence** | **Lab confirmed Influenza A incidence** | **Extrapolated Influenza A incidence** |
| --- | --- | --- | --- | --- | --- | --- | --- | --- | --- |
| 2017/18 | 0-5m | Lothian | 5923.6 (5437.5 - 6421) | 124.3 (56.5 - 214.8) | NA (NA - NA) | 1865.2 (1582.6 - 2159.2) | NA (NA - NA) | 271.3 (169.6 - 384.4) | NA (NA - NA) |
| 2017/18 | 0-5m | Other health boards | 7498.4 (7238.5 - 7734.8) | 105.3 (74.9 - 138.1) | 192.1 (154.4 - 234) | 1661.6 (1537.6 - 1781.1) | 2316.2 (2171.7 - 2462.1) | 142.8 (107.7 - 177.9) | 321.7 (271.5 - 379.1) |
| 2018/19 | 0-5m | Lothian | 7117.8 (6602.4 - 7656.3) | 386.3 (257.6 - 515.1) | NA (NA - NA) | 2481.9 (2153.8 - 2809.9) | NA (NA - NA) | 199 (117.1 - 304.4) | NA (NA - NA) |
| 2018/19 | 0-5m | Other health boards | 7893.4 (7613.2 - 8147.4) | 102.1 (71.2 - 135.3) | 398.5 (339.5 - 455.8) | 1583.4 (1471.8 - 1709.3) | 2867.9 (2718.2 - 3019.7) | 166.2 (128.2 - 201.8) | 213.6 (175.7 - 256.4) |
| 2019/20 | 0-5m | Lothian | 7633.9 (7045.7 - 8222.3) | 294.1 (171.5 - 416.6) | NA (NA - NA) | 3308.4 (2928.6 - 3713.1) | NA (NA - NA) | 245.1 (147 - 355.3) | NA (NA - NA) |
| 2019/20 | 0-5m | Other health boards | 7643.2 (7387.5 - 7889.2) | 59.6 (37.2 - 81.9) | 258.6 (211 - 302.9) | 2301.2 (2149.6 - 2450.2) | 3230.4 (3058.3 - 3413.3) | 163.8 (124.1 - 203.6) | 275.8 (225.9 - 332.6) |
| 2020/21 | 0-5m | Lothian | 2173.9 (1858.1 - 2489.7) | ^[5]^ | NA (NA - NA) | * | NA (NA - NA) | * | NA (NA - NA) |
| 2020/21 | 0-5m | Other health boards | 2111 (1973.1 - 2246.1) | 10.4 (2.6 - 20.8) | ^[6]^ | 2.6 (0 - 7.8) | † | 2.6 (0 - 10.4) | † |
| 2021/22 | 0-5m | Lothian | 10532.7 (9874.1 - 11191.3) | 633.9 (463.2 - 804.9) | NA (NA - NA) | 3718.2 (3315.9 - 4120.4) | NA (NA - NA) | 12.2 (0 - 36.6) | NA (NA - NA) |
| 2021/22 | 0-5m | Other health boards | 10187.8 (9897.9 - 10464.8) | 174.4 (133.4 - 218) | 673.8 (595.1 - 756.7) | 2021.1 (1882.6 - 2172.5) | 3364.4 (3183 - 3539.6) | 43.6 (23.1 - 66.7) | 54.9 (33.3 - 79.5) |
| 2022/23 | 0-5m | Lothian | 5558.3 (5045.1 - 6046.6) | 25 (0 - 62.6) | NA (NA - NA) | 1865.3 (1589.6 - 2166.1) | NA (NA - NA) | 75.1 (25 - 137.7) | NA (NA - NA) |
| 2022/23 | 0-5m | Other health boards | 5556.3 (5307.7 - 5786.7) | 31.4 (15.7 - 49.7) | 32.7 (15.7 - 52.3) | 2174.9 (2033.5 - 2324.1) | 1881.4 (1737.8 - 2015.2) | 99.5 (70.7 - 130.9) | 95.8 (65.4 - 128.2) |
| 2017/18 | 6m-1y | Lothian | 8603.7 (8021.9 - 9140.7) | 391.6 (257.3 - 514.7) | NA (NA - NA) | 1398.5 (1152.1 - 1633.5) | NA (NA - NA) | 290.9 (179 - 402.8) | NA (NA - NA) |
| 2017/18 | 6m-1y | Other health boards | 14925.9 (14592.9 - 15267.9) | 198.3 (159.6 - 239.3) | 698.5 (620 - 781.9) | 1370 (1255.9 - 1479.4) | 2404 (2258.9 - 2553.1) | 328.2 (278 - 378.4) | 491.3 (424 - 553.9) |
| 2018/19 | 6m-1y | Lothian | 10787.7 (10191.9 - 11450.7) | 606.8 (449.5 - 764.4) | NA (NA - NA) | 2595.8 (2269.9 - 2932.9) | NA (NA - NA) | 752.9 (584.3 - 943.9) | NA (NA - NA) |
| 2018/19 | 6m-1y | Other health boards | 14390.6 (14048.1 - 14723.8) | 184 (144.4 - 226) | 828.3 (747.8 - 908.6) | 1362.9 (1253.3 - 1465.4) | 3427.8 (3254.5 - 3597.1) | 512.5 (447.3 - 575.4) | 984.6 (889.9 - 1076.3) |
| 2019/20 | 6m-1y | Lothian | 9740.4 (9132.4 - 10395.2) | 339.1 (233.9 - 467.7) | NA (NA - NA) | 2841.4 (2490.6 - 3203.9) | NA (NA - NA) | 374.2 (257.2 - 514.5) | NA (NA - NA) |
| 2019/20 | 6m-1y | Other health boards | 13780.1 (13426.6 - 14107.6) | 162.6 (124.9 - 202.6) | 451 (384.1 - 518.4) | 2014.7 (1875.6 - 2153.7) | 3982.3 (3796.1 - 4166.1) | 433.6 (367.6 - 502) | 596.1 (527.8 - 669.2) |
| 2020/21 | 6m-1y | Lothian | 4641.8 (4176.4 - 5107.5) | 24.5 (0 - 61.2) | NA (NA - NA) | * | NA (NA - NA) | * | NA (NA - NA) |
| 2020/21 | 6m-1y | Other health boards | 6827.8 (6580.6 - 7060.2) | 22.2 (9.9 - 37.1) | 62.9 (39.6 - 86.5) | 9.9 (2.5 - 19.8) | † | 2.5 (0 - 7.4) | † |
| 2021/22 | 6m-1y | Lothian | 12432.9 (11729.6 - 13148.2) | 1013.2 (810.6 - 1227.8) | NA (NA - NA) | 2670.2 (2312.6 - 3015.9) | NA (NA - NA) | 47.7 (11.9 - 95.4) | NA (NA - NA) |
| 2021/22 | 6m-1y | Other health boards | 15769.6 (15394.9 - 16136.6) | 438.3 (369.5 - 502.1) | 1241.6 (1129 - 1343.1) | 1524 (1411.8 - 1641.2) | 3421.6 (3256.9 - 3616.2) | 86.6 (58.6 - 117.3) | 116.7 (84.1 - 152.9) |
| 2022/23 | 6m-1y | Lothian | 8766.3 (8149.5 - 9395) | 296.6 (189.5 - 415.2) | NA (NA - NA) | 1755.6 (1470.9 - 2028.8) | NA (NA - NA) | 320.3 (201.7 - 450.8) | NA (NA - NA) |
| 2022/23 | 6m-1y | Other health boards | 9669.1 (9367 - 9981.3) | 119.9 (84.9 - 154.8) | 390.4 (329.6 - 449.6) | 1700.6 (1578.2 - 1823) | 1899.7 (1767.9 - 2045.2) | 379.6 (319.6 - 439.5) | 410.1 (347 - 472) |
| 2017/18 | 2-4y | Lothian | 1389.8 (1265.4 - 1524.9) | 74.6 (46.2 - 106.7) | NA (NA - NA) | 110.2 (74.6 - 149.4) | NA (NA - NA) | 67.5 (39.1 - 99.5) | NA (NA - NA) |
| 2017/18 | 2-4y | Other health boards | 2522.1 (2441.6 - 2604) | 34.5 (24.4 - 43.8) | 141.1 (120 - 161.7) | 140.2 (121.4 - 159.6) | 217.3 (194.1 - 243.7) | 78.3 (64 - 92.7) | 114.6 (96.3 - 132.3) |
| 2018/19 | 2-4y | Lothian | 1635 (1501.1 - 1786.9) | 115.7 (79.6 - 155.6) | NA (NA - NA) | 253.2 (191.7 - 314.7) | NA (NA - NA) | 220.6 (166.4 - 278.5) | NA (NA - NA) |
| 2018/19 | 2-4y | Other health boards | 2698.1 (2608.2 - 2789.5) | 34.3 (24.8 - 44.6) | 178.6 (157.1 - 201.7) | 130.1 (112.5 - 149.1) | 355.9 (324.5 - 388.8) | 181.2 (157.1 - 203.2) | 381 (348.6 - 412.9) |
| 2019/20 | 2-4y | Lothian | 1767.2 (1604.5 - 1926.1) | 70.2 (40.7 - 103.5) | NA (NA - NA) | 195.9 (144.2 - 251.4) | NA (NA - NA) | 125.7 (84.9 - 166.4) | NA (NA - NA) |
| 2019/20 | 2-4y | Other health boards | 2472.6 (2386 - 2556.2) | 30.1 (21.1 - 40.6) | 102.5 (86.5 - 121.2) | 193.4 (170.9 - 217.5) | 285.2 (256.7 - 312.4) | 113.7 (95.6 - 131.7) | 187.1 (165.6 - 210) |
| 2020/21 | 2-4y | Lothian | 674.8 (578.9 - 782.1) | 3.8 (0 - 11.5) | NA (NA - NA) | 3.8 (0 - 11.5) | NA (NA - NA) | * | NA (NA - NA) |
| 2020/21 | 2-4y | Other health boards | 1210.3 (1150 - 1274.3) | 5.4 (1.5 - 9.3) | 12.1 (6.9 - 19.3) | 2.3 (0 - 5.4) | 8.2 (3.9 - 13.1) | 3.1 (0.8 - 6.2) | † |
| 2021/22 | 2-4y | Lothian | 2640.8 (2461 - 2840.1) | 234.4 (175.8 - 293.1) | NA (NA - NA) | 453.2 (382.7 - 535.2) | NA (NA - NA) | 39.1 (15.6 - 62.5) | NA (NA - NA) |
| 2021/22 | 2-4y | Other health boards | 3380 (3288.9 - 3473.5) | 95.8 (79.3 - 113.9) | 301.2 (271 - 331.5) | 304.8 (274.1 - 334.6) | 572.9 (532.5 - 612.7) | 40.8 (29.8 - 51.8) | 64.9 (50.3 - 79.3) |
| 2022/23 | 2-4y | Lothian | 1681.8 (1519 - 1844.6) | 77.5 (46.5 - 116.3) | NA (NA - NA) | 244.1 (186 - 306.1) | NA (NA - NA) | 112.4 (73.6 - 155) | NA (NA - NA) |
| 2022/23 | 2-4y | Other health boards | 2372.4 (2289.6 - 2456.8) | 26 (17.4 - 35.5) | 105.2 (87.5 - 123.8) | 244.5 (215.3 - 271.3) | 334.4 (303.6 - 363.6) | 153.8 (133.3 - 174.3) | 182.2 (159.3 - 205.1) |
| 2017/18 | <5y | Lothian | 3667.5 (3491 - 3839.5) | 145.9 (111.1 - 185.1) | NA (NA - NA) | 699.1 (622.8 - 773.1) | NA (NA - NA) | 150.3 (115.4 - 187.3) | NA (NA - NA) |
| 2017/18 | <5y | Other health boards | 5874.7 (5776.8 - 5976.6) | 79.7 (67.8 - 91.3) | 259.1 (238.3 - 280.9) | 667.2 (635.3 - 702.6) | 1039.5 (998.1 - 1081.8) | 139.1 (124 - 155.1) | 227 (207.3 - 246.8) |
| 2018/19 | <5y | Lothian | 4480.2 (4289.4 - 4664.4) | 263.9 (217.4 - 310.5) | NA (NA - NA) | 1137.8 (1040.2 - 1235.4) | NA (NA - NA) | 321.6 (272.8 - 381.5) | NA (NA - NA) |
| 2018/19 | <5y | Other health boards | 5946.3 (5850.7 - 6041) | 76.2 (64.9 - 87.4) | 346 (322.2 - 369.6) | 644.5 (610.7 - 678.7) | 1427.1 (1376.4 - 1474.2) | 242.5 (221.3 - 262.3) | 466 (439.9 - 492.6) |
| 2019/20 | <5y | Lothian | 4419.4 (4234.2 - 4618.2) | 164.5 (130.2 - 205.7) | NA (NA - NA) | 1293.4 (1186 - 1407.6) | NA (NA - NA) | 196.5 (153.1 - 239.9) | NA (NA - NA) |
| 2019/20 | <5y | Other health boards | 5664.7 (5572.9 - 5758.4) | 61.7 (51.5 - 71.9) | 200.3 (182.3 - 218.5) | 945.8 (905 - 983.9) | 1563.3 (1509 - 1614.2) | 186 (167.9 - 206) | 284.2 (261.6 - 307.6) |
| 2020/21 | <5y | Lothian | 1727.8 (1614.7 - 1852.5) | 8.8 (0 - 20.4) | NA (NA - NA) | 3.8 (0 - 15.3) | NA (NA - NA) | * | NA (NA - NA) |
| 2020/21 | <5y | Other health boards | 2466.5 (2403.1 - 2530.8) | 9.6 (5.8 - 13.9) | 21.6 (15.8 - 28.3) | 3.8 (1.4 - 6.7) | 7.5 (4.3 - 11) | 2.9 (1 - 5.3) | † |
| 2021/22 | <5y | Lothian | 6122.3 (5892.3 - 6357) | 466.9 (405.3 - 526.3) | NA (NA - NA) | 1528.8 (1407.9 - 1644.9) | NA (NA - NA) | 35.6 (19 - 54.5) | NA (NA - NA) |
| 2021/22 | <5y | Other health boards | 7036.7 (6918.9 - 7142.9) | 176.1 (158.1 - 193.6) | 551.4 (519.6 - 584.8) | 863.1 (827.1 - 904.5) | 1646.3 (1595.3 - 1701.4) | 50.1 (40.9 - 60.3) | 72.9 (60.8 - 84.2) |
| 2022/23 | <5y | Lothian | 3829.6 (3640 - 4028.5) | 111.3 (80.5 - 142.1) | NA (NA - NA) | 852.6 (767.3 - 940.2) | NA (NA - NA) | 146.8 (111.3 - 187.1) | NA (NA - NA) |
| 2022/23 | <5y | Other health boards | 4390.8 (4309.3 - 4484.9) | 45.4 (36.6 - 54.1) | 147.4 (131.7 - 162.4) | 888.6 (850 - 926.6) | 928.4 (886.6 - 968.6) | 187.8 (169.2 - 206.8) | 210.6 (190.2 - 230.7) |

# Supplementary Table S7: Findings of sensitivity analysis conducted by limiting to episodes with specimens collected for viral testing between 3 days before and 3 days after the date of admission in older adults in Lothian by age bands and seasons

| **Season** | **Age band** | **Lab-confirmed hMPV hospital incidence (sensitivity analysis)** | **Lab-confirmed hMPV hospital incidence (main analysis)** | **Lab-confirmed RSV hospital incidence (sensitivity analysis)** | **Lab-confirmed RSV hospital incidence (main analysis)** | **Lab-confirmed Influenza A hospital incidence (sensitivity analysis)** | **Lab-confirmed Influenza A hospital incidence (main analysis)** |
| --- | --- | --- | --- | --- | --- | --- | --- |
| 2017/18 | 0-5m | 124.3 (56.5 - 203.5) | 124.3 (56.5 - 214.8) | 1831.3 (1571.3 - 2102.9) | 1865.2 (1582.6 - 2159.2) | 237.4 (135.7 - 339.1) | 271.3 (169.6 - 384.4) |
| 2018/19 | 0-5m | 386.3 (257.6 - 526.8) | 386.3 (257.6 - 515.1) | 2435 (2107.2 - 2739.4) | 2481.9 (2153.8 - 2809.9) | 199 (105.4 - 304.4) | 199 (117.1 - 304.4) |
| 2019/20 | 0-5m | 281.8 (171.5 - 392.1) | 294.1 (171.5 - 416.6) | 3210.4 (2830.2 - 3614.8) | 3308.4 (2928.6 - 3713.1) | 245.1 (147 - 355.3) | 245.1 (147 - 355.3) |
| 2020/21 | 0-5m | ^[7]^ | * | * | * | * | * |
| 2021/22 | 0-5m | 633.9 (463.2 - 816.8) | 633.9 (463.2 - 804.9) | 3681.6 (3279.3 - 4071.7) | 3718.2 (3315.9 - 4120.4) | 12.2 (0 - 36.6) | 12.2 (0 - 36.6) |
| 2022/23 | 0-5m | 25 (0 - 62.6) | 25 (0 - 62.6) | 1852.8 (1577.1 - 2153.2) | 1865.3 (1589.6 - 2166.1) | 75.1 (25 - 137.7) | 75.1 (25 - 137.7) |
| 2017/18 | 6m-1y | 358 (223.8 - 492.3) | 391.6 (257.3 - 514.7) | 1387.3 (1163.6 - 1633.8) | 1398.5 (1152.1 - 1633.5) | 279.7 (179 - 391.6) | 290.9 (179 - 402.8) |
| 2018/19 | 6m-1y | 584.3 (427 - 752.9) | 606.8 (449.5 - 764.4) | 2573.3 (2269.9 - 2910.7) | 2595.8 (2269.9 - 2932.9) | 707.9 (528.1 - 887.7) | 752.9 (584.3 - 943.9) |
| 2019/20 | 6m-1y | 339.1 (233.9 - 467.7) | 339.1 (233.9 - 467.7) | 2806.4 (2455.6 - 3157.4) | 2841.4 (2490.6 - 3203.9) | 350.8 (222.2 - 479.4) | 374.2 (257.2 - 514.5) |
| 2020/21 | 6m-1y | 24.5 (0 - 61.2) | 24.5 (0 - 61.2) | * | * | * | * |
| 2021/22 | 6m-1y | 1013.2 (798.7 - 1227.8) | 1013.2 (810.6 - 1227.8) | 2622.5 (2288.7 - 2980.1) | 2670.2 (2312.6 - 3015.9) | 47.7 (11.9 - 95.4) | 47.7 (11.9 - 95.4) |
| 2022/23 | 6m-1y | 284.7 (177.9 - 403.3) | 296.6 (189.5 - 415.2) | 1743.8 (1482.8 - 2028.5) | 1755.6 (1470.9 - 2028.8) | 320.3 (201.7 - 450.8) | 320.3 (201.7 - 450.8) |
| 2017/18 | 2-4y | 71.1 (42.7 - 103.1) | 74.6 (46.2 - 106.7) | 106.6 (71.1 - 149.3) | 110.2 (74.6 - 149.4) | 67.5 (39.1 - 99.5) | 67.5 (39.1 - 99.5) |
| 2018/19 | 2-4y | 104.9 (68.7 - 148.3) | 115.7 (79.6 - 155.6) | 246 (184.5 - 307.5) | 253.2 (191.7 - 314.7) | 220.6 (166.4 - 278.5) | 220.6 (166.4 - 278.5) |
| 2019/20 | 2-4y | 70.2 (40.7 - 103.5) | 70.2 (40.7 - 103.5) | 192.2 (140.5 - 247.7) | 195.9 (144.2 - 251.4) | 125.7 (84.9 - 166.4) | 125.7 (84.9 - 166.4) |
| 2020/21 | 2-4y | 3.8 (0 - 11.5) | 3.8 (0 - 11.5) | 3.8 (0 - 15.3) | 3.8 (0 - 11.5) | * | * |
| 2021/22 | 2-4y | 230.5 (171.9 - 289.1) | 234.4 (175.8 - 293.1) | 445.3 (371.1 - 531.3) | 453.2 (382.7 - 535.2) | 39.1 (15.6 - 62.5) | 39.1 (15.6 - 62.5) |
| 2022/23 | 2-4y | 73.6 (42.6 - 108.5) | 77.5 (46.5 - 116.3) | 240.3 (186 - 298.4) | 244.1 (186 - 306.1) | 112.4 (73.6 - 155) | 112.4 (73.6 - 155) |
| 2017/18 | <5y | 137.2 (102.4 - 172) | 145.9 (111.1 - 185.1) | 688.2 (616.3 - 766.6) | 699.1 (622.8 - 773.1) | 104.6 (65.9 - 143.4) | 150.3 (115.4 - 187.3) |
| 2018/19 | <5y | 252.8 (208.5 - 301.6) | 263.9 (217.4 - 310.5) | 1120.1 (1024.6 - 1222.1) | 1137.8 (1040.2 - 1235.4) | 141.6 (106.7 - 176.4) | 321.6 (272.8 - 381.5) |
| 2019/20 | <5y | 162.2 (128 - 201.1) | 164.5 (130.2 - 205.7) | 1265.9 (1160.8 - 1368.8) | 1293.4 (1186 - 1407.6) | 310.5 (259.5 - 361.5) | 196.5 (153.1 - 239.9) |
| 2020/21 | <5y | 8.8 (0 - 20.4) | 8.8 (0 - 20.4) | 3.8 (0 - 11.5) | 3.8 (0 - 15.3) | 189.7 (150.8 - 233.1) | * |
| 2021/22 | <5y | 464.6 (402.9 - 528.6) | 466.9 (405.3 - 526.3) | 1507.5 (1396 - 1623.7) | 1528.8 (1407.9 - 1644.9) | 35.6 (19 - 54.5) | 35.6 (19 - 54.5) |
| 2022/23 | <5y | 106.6 (78.2 - 137.4) | 111.3 (80.5 - 142.1) | 845.5 (757.9 - 930.8) | 852.6 (767.3 - 940.2) | 142.1 (106.6 - 180) | 146.8 (111.3 - 187.1) |

# Supplementary Table S8: Characteristics of all-cause RTI, hMPV, RSV, and Influenza A hospital episodes in older adults in Scotland (2017-2023)

|  |  | **All-cause RTI**  **(n = 77,847)** | **hMPV**  **(n = 1,462)** | **RSV**  **(n = 10,985)** | **Influenza A (n = 2,124)** |
| --- | --- | --- | --- | --- | --- |
| **Age** | 0-5m | 19,757  (25.38%) | 318  (21.75%) | 5,025  (45.74%) | 321  (15.11%) |
|  | 6m-1y | 36,118  (46.40%) | 694  (47.47%) | 4,295  (39.10%) | 891  (41.95%) |
|  | 2-4y | 21,972  (28.22) | 450  (30.78%) | 1,665  (15.16%) | 912  (42.94%) |
| **Sex** | Female | 32,670  (41.97%) | 615  (42.07%) | 4,867  (44.31%) | 949  (44.68%) |
|  | Male | 45,177  (58.03%) | 847  (57.93%) | 6,118  (55.69%) | 1,175  (55.32%) |
| **Ethnicity** | White | 199,926  (86.36%) | 1,085  (74.21%) | 7,541  (68.65%) | 1,524  (71.75%) |
|  | Non-White | 29,593  (12.78%) | 350  (23.94%) | 2,620  (23.85%) | 471  (22.18%) |
|  | Data unavailable | 1,989  (0.86%) | 27  (1.85%) | 824  (7.50%) | 129  (6.07%) |
| **SIMD ^[8]^** | 1 | 21,853  (28.07%) | 319  (21.82%) | 2.936  (26.73%) | 621  (29.24%) |
|  | 2 | 16,819  (21.61%) | 321  (21.96%) | 2,335  (21.26%) | 478  (22.50%) |
|  | 3 | 13,685  (17.58%) | 288  (19.70%) | 1.986  (18.08%) | 364  (17.14%) |
|  | 4 | 13,923  (17.89%) | 275  (18.81%) | 2,036  (18.53%) | 354  (16.67%) |
|  | 5 | 111,41  (14.31%) | 255  (17.44%) | 1,641  (14.94%) | 296  (13.94%) |
|  | Data unavailable | 426  (0.55%) | 4  (0.27%) | 51  (0.46%) | 11  (0.52%) |

# Supplementary Table S9: Number and proportion of laboratory-confirmed hMPV, RSV and Influenza A RTI episodes requiring long hospital length of stay (LOS) in children aged <5 years in Scotland by age bands during six annual seasons (2017-2023)

| **Age bands** | **Region** | **Laboratory-confirmed hMPV RTI hospital episodes requiring long LOS** | **Laboratory-confirmed RSV RTI hospital episodes requiring long LOS** | **Laboratory-confirmed Influenza A RTI hospital episodes requiring long LOS** |
| --- | --- | --- | --- | --- |
| **0-5 months** | Lothian | 16 (13.1%) | 133 (12.1%) | 5 (7.4%) |
|  | Other health boards | 20 (10.2%) | 344 (8.8%) | 7 (2.8%) |
|  | Overall (Scotland) | 36 (11.3%) | 477 (9.5%) | 12 (3.7%) |
| **6 months – 1 year** | Lothian | 35 (15.2%) | 83 (8.5%) | 12 (7.7%) |
|  | Other health boards | 35 (7.5%) | 183 (5.5%) | 31 (4.2%) |
|  | Overall (Scotland) | 70 (10.1%) | 266 (6.2%) | 43 (4.8%) |
| **2 – 4 years** | Lothian | 19 (15.0%) | 30 (9.0%) | 6 (3.9%) |
|  | Other health boards | 19 (6.4%) | 93 (7.0%) | 39 (5.1%) |
|  | Overall (Scotland) | 38 (8.4%) | 123 (7.4%) | 45 (4.9%) |
| **Overall (<5 years)** | Lothian | 70 (13.9%) | 246 (10.0%) | 23 (6.1%) |
|  | Other health boards | 74 (7.7%) | 620 (7.2%) | 77 (4.4%) |
|  | Overall (Scotland) | 98 (9.8%) | 866 (7.9%) | 100 (4.7%) |

# Supplementary Table S10: ICU admission rate and in-hospital and post-discharge CFR associated with laboratory-confirmed hMPV, RSV, and Influenza A hospital admissions

|  | **Laboratory-confirmed hMPV RTI hospital episodes** | **Laboratory-confirmed RSV RTI hospital episodes** | **Laboratory-confirmed Influenza A hospital episodes** |
| --- | --- | --- | --- |
| **ICU admission rate** | 0.34 (0.07 – 0.68) | 0.12 (0.06 – 0.18) | 0.05 (0 – 0.19) |
| **In-hospital CFR** | 0.14 (0 – 0.34) | 0.01 (0 – 0.03) | 0 (0 – 0) |
| **90 days post-discharge CFR** | 0.34 (0.07 – 0.68) | 0.05 (0.02 – 0.10) | 0.05 (0 – 0.14) |

1. The Chi-square test showed a statistically significant distribution in the age distribution between all-cause RTIs in Lothian and other health boards (χ²=26.75, p<0.001)
2. The Chi-square test showed a statistically significant distribution in the ethnicity distribution between all-cause RTIs in Lothian and other health boards (χ²=2053.51, p<0.001)
3. The Chi-square test showed a statistically significant distribution in the SIMD distribution between all-cause RTIs in Lothian and other health boards (χ²=5317.39, p<0.001)
4. * There were no laboratory-confirmed episodes in Lothian in the age band and season combination. As a result, extrapolation was not possible.
5. There were no laboratory-confirmed episodes in Lothian in the age band and season combination.
6. There were no laboratory-confirmed episodes in Lothian in the age band and season combination. As a result, extrapolation was not possible.
7. There were no lab-confirmed cases in Lothian in the age band and season combination.
8. SIMD ranks data zones in Scotland in order of deprivation. The data are presented as SIMD quintiles with 1 indicating the most deprived area and higher quintiles progressively representing the less deprived areas
